# Live Cell Imaging by Single-Shot Common-Path Wide Field-of-View Reflective Digital Holographic Microscope

**DOI:** 10.3390/s24030720

**Published:** 2024-01-23

**Authors:** Manoj Kumar, Takashi Murata, Osamu Matoba

**Affiliations:** 1 Department of Systems Science, Graduate School of System Informatics, Kobe University, Rokkodai 1-1, Nada, Kobe 657-8501, Japan; 2 Center of Optical Scattering Image Science, Kobe University, Rokkodai 1-1, Nada, Kobe 657-8501, Japan; 3 Department of Applied Bioscience, Kanagawa Institute of Technology, Atsugi 243-0292, Japan

**Keywords:** quantitative phase imaging, digital holographic microscopy, live cell imaging

## Abstract

Quantitative phase imaging by digital holographic microscopy (DHM) is a nondestructive and label-free technique that has been playing an indispensable role in the fields of science, technology, and biomedical imaging. The technique is competent in imaging and analyzing label-free living cells and investigating reflective surfaces. Herein, we introduce a new configuration of a wide field-of-view single-shot common-path off-axis reflective DHM for the quantitative phase imaging of biological cells that leverages several advantages, including being less-vibration sensitive to external perturbations due to its common-path configuration, also being compact in size, simple in optical design, highly stable, and cost-effective. A detailed description of the proposed DHM system, including its optical design, working principle, and capability for phase imaging, is presented. The applications of the proposed system are demonstrated through quantitative phase imaging results obtained from the reflective surface (USAF resolution test target) as well as transparent samples (living plant cells). The proposed system could find its applications in the investigation of several biological specimens and the optical metrology of micro-surfaces.

## 1. Introduction

In the field of imaging and optical metrology, phase retrieval is the key research objective because it provides the morphology and topography of the object under investigation. The local optical properties of the object, including thickness, refractive index, etc., can be retrieved by the quantitative evaluation of the phase of light waves passing through the phase object (i.e., a (semi) transparent object that does not absorb or scatter light significantly). Digital holographic microscopy (DHM) [1,2] arises from the application of digital holography to microscopy, and is a powerful and well-known quantitative phase imaging technique that works in a noninvasive and label-free manner, and provides the sample’s structural information by exploiting the phase information of the object wave from the sample. The direct access to quantitative phase information makes it a true three-dimensional (3D) imaging technique, and it has become an essential medical diagnosis tool for investigating blood cells [3,4], neuronal cells [5], cancer cell research [6], fibroblast cells [7], testate amoeba [8], diatom skeletons [9], infections [10], cell culture quality control [11], lung cancer cells [12], plant cells [13], and several other applications [14].

According to the type of object under investigation (transparent or opaque/reflective), DHM systems can be further classified into two configurations: transmission and reflection modes. The transmission-mode DHM system is used for the investigation of (semi) transparent objects, in which light transilluminates the phase object and reaches the image sensor together with the reference wave; their interference (digital hologram) is recorded by the image sensor. Unlike the transmission-mode DHM, in reflection-mode DHM, the object beam, after reflecting or scattering from the object, reaches the image sensor where it interferes with the reference beam to make a digital hologram. Transmission-mode DHMs have been used for the investigation of various types of (semi) transparent biological cells, as stated in [3,4,5,6,7,8,9,10,11,12,13,14,15]. On the other hand, reflection-mode DHMs have been used for the characterization and topography detection of engineered surfaces such as the silicon surface of an integrated circuit [15], microstructures fabricated in bulk lithium niobate [16], semiconductor etching [17], analysis of MEMS and MOEMS [18,19], microlens [20], etc. The benefits of DHM have been exploited by adopting different optical designs, namely, in-line and off-axis DHMs. The off-axis scheme of a DHM is an ideal tool for various investigations with several advantages over an in-line DHM, such as twin-image free reconstruction; however, it is at the cost of reduced precision and the high space-bandwidth-product of the image sensor. Furthermore, the traditional off-axis scheme of DHM suffers from poor phase stability due to the two-channel configuration, limiting its use for some applications, for instance, in measuring small cell thickness fluctuations. In recent years, various schemes of common-path DHMs have been devised to overcome this issue [21,22,23,24,25,26,27,28,29,30] in conjunction with providing other advantages such as a compact optical setup, less vibration sensitivity to external perturbations, and high mechanical stability and sensitivity. In such systems, both the object and reference beams follow the same path and pass through the same optical elements.

In recent years, inspections of micro/nano-scale three-dimensional (3D) topographic profiles of opaque/reflective objects have gained great interest in the realms of science, technology, and manufacturing. The use of reflection-mode DHM for the characterization of opaque micro/nano-surfaces started with the pioneering work of Cuche et al. [31]. Afterward, several investigations with different configurations of reflection-mode DHMs based on modified Mach–Zehnder [16,32,33], Michelson [31,34,35,36], Twyman–Green [37], Sagnac [38], modified Fizeau [39,40,41] interferometers have been reported. However, most of these investigations have been implemented by utilizing two-channel interferometer configuration-based DHM systems with lower sensitivity and stability, bulky and complex optical designs, expensive, and more prone to environmental disturbances. Therefore, common-path configurations of DHM systems (such as Refs. [21,22,23,24,25,26,27,28,29,30]) are of particular interest as they are free from these drawbacks. Recently, several common-path reflection-mode DHMs [17,20,42,43,44,45,46,47] have been reported to enhance performance in terms of simplicity, robustness, compactness, and stability. Furthermore, common-path DHMs, depending on generating the reference beam, can be of different types, including the self-interference common-path DHMs [48,49] and specific optical element-based common-path DHMs [4,21,26,50,51]. Common-path DHM systems based on specific optical elements require special optical components, difficult optical designs, and sometimes a very high degree of precision, especially to generate a clean reference beam by filtering one beam with a pinhole at its Fourier plane to erase all object information. On the other hand, self-interference common-path DHMs suffer from several limitations, including reduced FOV, irrelevant for dynamic object investigations, and applicability for sparse objects. For instance, Jiwei Zhang et al. [42] proposed a highly stable (temporal stability of 0.071 rad) common-path DHM based on prism-coupling surface plasmon resonance for near-field phase imaging. However, in this work, the reference beam is generated from a portion of the object beam that does not carry object information. This DHM system is useful for several applications but limits its applications in certain investigations, such as being appropriate for only sparse samples. Furthermore, this DHM system has reduced the overall FOV as the object information is present only in a small portion of the object beam. 

Aimed at addressing the aforementioned drawbacks of DHMs and inspired by our previous works [39,40,41], in this paper, a single-shot wide field-of-view (FOV) common-path off-axis DHM, operating in reflection mode for quantitative phase imaging of (semi) transparent and reflective objects, is developed. The main advantages of the proposed DHM system, making it different from the other reported DHM systems, are common-path reflection mode configuration, an extremely simple and compact design, single-shot recording, and retrieval of complete object information (phase and amplitude) free from spherical aberrations over the entire FOV, with higher phase stability (>43 times) compared to its counterparts [13]. It is also applicable for the quantitative phase imaging of transparent as well as the topography of reflective samples. The optical layout of the proposed DHM system is extremely simple, based on a plate beam splitter for both illuminating the sample in reflection and generating the digital holograms. The plate beam splitter is employed just before the microscope objective. The reflected beam from the plate beam splitter serves as the clean reference beam, and the transmitted beam (serving as the object beam) illuminates the specimen and traverses back the same path. The object and reference beams travel the same path, and their interference is recorded by the image sensor. The proposed system enables wide FOV quantitative phase imaging and topography analysis of reflective/opaque as well as (semi) transparent objects using a single-shot operation principle.

## 2. Materials and Methods

**Figure 1:**
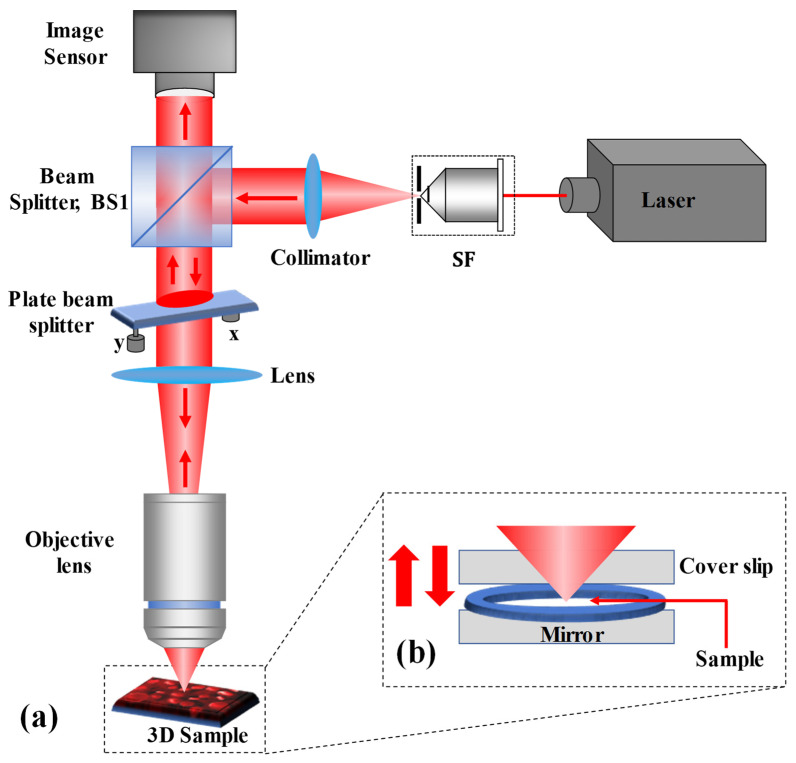
(**a**) Schematic of the experimental setup of common-path reflective DHM. (**b**) Scheme for the preparation of (semi) transparent specimens.

The proposed single-shot wide FOV common-path off-axis reflection-mode DHM, based on a plate beam splitter, is schematically depicted in Figure 1a. After reflecting by a 50:50 cube beam splitter (BS1), the collimated He-Ne (wavelength 632.8 nm) laser beam is sent directly upward through the object under study. A lens (of focal length, *f* = 50 mm) focuses the reflected collimated beam from BS1 on the microscope objective. The plate beam splitter (Thorlabs BSN10R, 25 × 36 mm^2^ 10:90 (R:T)) is practically oriented horizontally between BS1 and the lens, so that the incident laser beam is almost normal (with a small angle so as to achieve an off-axis interferometer and eliminate multiple reflected beams reaching the image sensor). The plate beam splitter is held in place by a two-adjustment kinematic mount. The plate beam splitter generates two beams: reflected 10% (reference beam) and transmitted 90% (object beam) beams. The transmitted beam from the plate beam splitter illuminates the reflective/opaque specimen or transilluminates the phase object. It should be noted that the coherence length of the laser source used in the experiment is 200 mm; therefore, the path difference between the object and reference is less than this coherence length, otherwise no fringe pattern will be observed between the object and reference beams. Furthermore, the phase object is plated on a mirrored surface [silver mirror (Thorlabs BB1-E02)] that reflects the forward diffraction back through the microscope objective, as depicted in Figure 1b. The reflected beam from the object goes back through the system, passes through the plate beam splitter and BS1, and reaches the image sensor (Sony Pregius IMX 264, sensor format: 2448 × 2048 pixels, pixel size of 3.45 μm), where it interferes with the reference beam to form a digital hologram. Therefore, the proposed DHM system provides a wide FOV equivalent to the physical size of the image sensor. The proposed system overcomes the limitations of the reduced FOV of self-referencing common-path DHMs [29,30,48,49], in which the reference beam is derived from a portion of the object beam. Additionally, it removes the issue of precise alignment and the need for specialized optical components in other common-path DHMs [4,21,26,50,51], in which the object beam is divided into two beams, where one of the beams is spatially filtered by a pinhole at its Fourier plane to generate a clean reference beam.

A single-shot holographic method, the principal component analysis (PCA)-based phase aberration compensation method, is adopted to recover the phase information accurately [52]. In this method, only a single digital hologram in the presence of the object is recorded. The phase distribution is decomposed into a set of values of uncorrelated variables, called principal components, and the aberration term is retrieved from the first principal component. Using singular value decomposition, the first principal component of the exponential term of the filtered hologram is measured. The linear and quadratic coefficients are identified using least-squares fitting, and their conjugates are multiplied with the filtered hologram to obtain the aberration-corrected phase distribution. The obtained phase is wrapped in the range (−π, π) radians corresponding to the principal value of the arctan function. An unwrapping algorithm is required to remove this phase discontinuity to obtain the continuous actual phase difference. The unwrapped phase provides useful information regarding the deformation, thickness, refractive index, etc., of the specimen. It should be noted that the measured phase value by the proposed system is twice the expected value, which should be divided by 2 to be consistent with its expected value.

## 3. Results

### 3.1. Phase Stability Measurement

**Figure 2:**
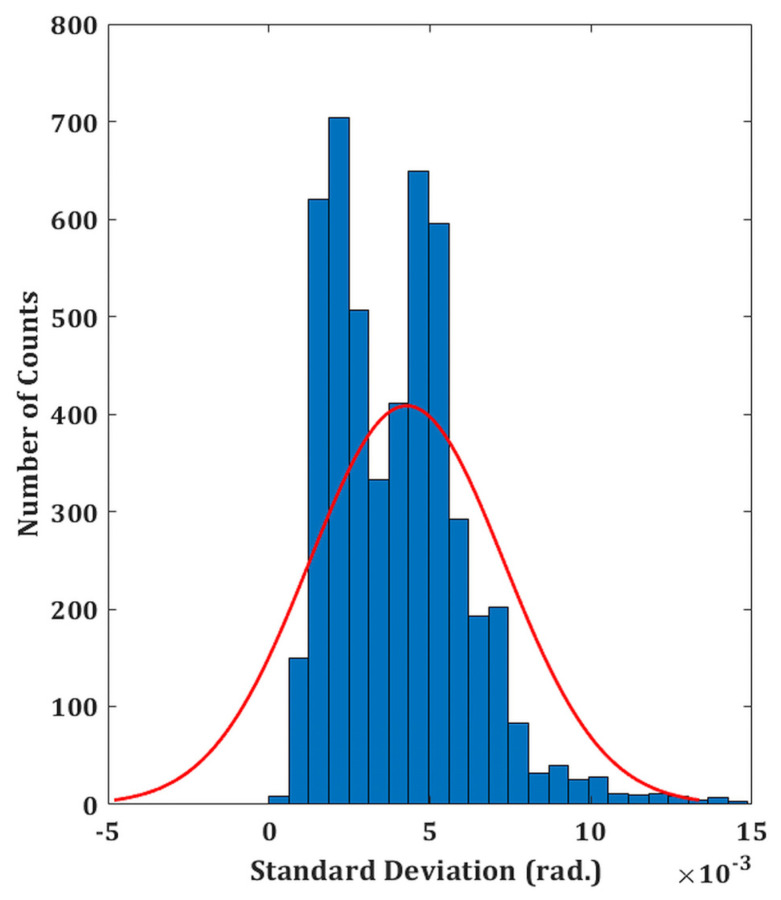
Temporal stability of the proposed setup. Histogram of the standard deviation of the reconstructed phase distributions for a defined spatial location. The red fitting curve appears to be approximately Gaussian type.

The experimental validity of the proposed DHM system is performed on both reflecting as well as transparent specimens. The temporal phase stability is first measured in order to assess the system’s performance. This involves recording a time series of holograms (5000 total) at a rate of 50 frames per second for 100 s without any vibration isolation. A holographic system’s capacity to withstand external mechanical vibrations or air turbulence is demonstrated by its better temporal stability. In comparison to the traditional two-channel architecture, common-path DHMs are anticipated to exhibit greater temporal stability, making them more resilient to air turbulence and external mechanical vibrations. The phase distributions are then extracted from the recorded digital holograms by numerically reconstructing each hologram. Comparing the extracted phase distributions to the phase of the first hologram yields the phase difference distributions. Since the standard deviation of the temporal variation of the phase distribution serves as the measure of fluctuation, the standard deviation is computed at each spatial location (150 × 150 pixel points) of every phase difference distribution [40]. The histogram of the standard deviation is shown in Figure 2, which indicates a temporal phase stability (mean fluctuation) of 0.0046 radians. The measured temporal stability of the proposed system is >43 times higher compared to a Mach–Zehnder-type DHM system (~0.2 radians), as reported in our previous work [21].

### 3.2. Inspection of Reflective Surfaces

The biological specimen (a phase object) is prepared on the silver mirror. An adhesive spacer is placed onto the mirror, and the biological specimen with distilled water is placed on the mirror inside the spacer, with a coverslip placed over the spacer creating a closed imaging chamber with a uniformly distributed layer of water, depicted in Figure 1b. There is no need to prepare a special arrangement for imaging the reflecting surfaces, such as a negative USAF resolution target.

**Figure 3:**
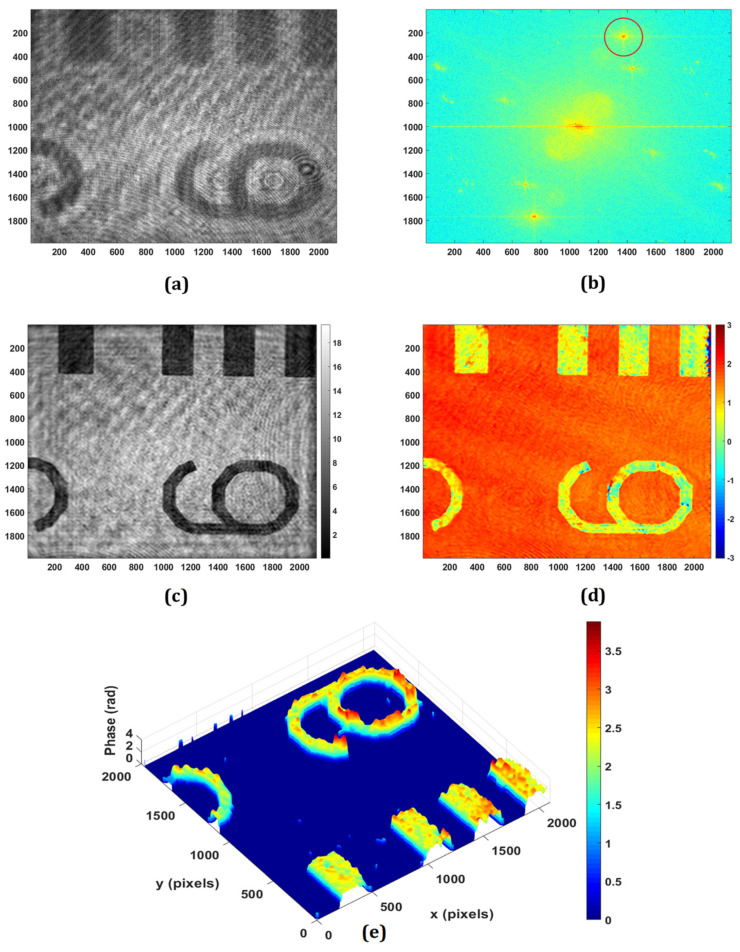
Experimental results of the negative USAF resolution test target: (**a**) recorded digital hologram; (**b**) Fourier transform of (**a**); (**c**) retrieved intensity; (**d**) 2D phase; (**e**) pseudo-3D phase map.

The application of the proposed DHM for retrieving the phase imaging of reflective/engineered surfaces is experimentally demonstrated. Figure 3 shows the experimental results obtained from the negative USAF resolution target with the use of a microscope objective of 20×, 0.4 NA. The minimum lateral (λ/NA) and axial ($2\lambda /NA^{2}$) resolutions of the system are 1.58 μm and 7.91 μm, respectively. Figure 3a,b show the recorded hologram and its Fourier spectrum, respectively. The PCA-based phase aberration compensation method is applied to the first-order spectrum (as depicted in the red circle in Figure 3b) to retrieve the amplitude and phase information. It should be noted that there are some other orders, as observed in the Fourier spectrum of the recorded digital hologram, due to multiple reflections from the plate beam splitter. Figure 3c,d depict the retrieved amplitude and wrapped phase at the focused plane, respectively, which is obtained at the reconstruction distance of 65 mm. The obtained wrapped phase is unwrapped using the PUMA phase unwrapping algorithm [53], and the rendered pseudo-3D representation of the unwrapped phase is shown in Figure 3e.

### 3.3. Testing of Micro-Optics

**Figure 4:**
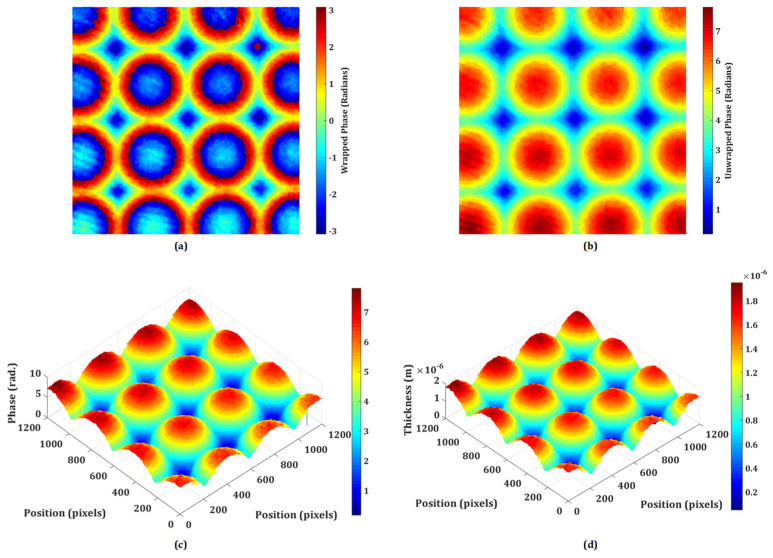
Experimental results of the micro-lenslet array: (**a**) retrieved wrapped phase, (**b**) unwrapped phase, (**c**) pseudo-3D unwrapped phase, and (**d**) 3D thickness profile. *x-* and *y*-axes: 1200 pixels × 1200 pixels.

DHM offers a powerful set of tools for the testing and characterizing micro-optics, providing high-resolution, quantitative, and non-destructive analysis. Its ability to capture dynamic processes and assess complex optical surfaces makes it a valuable technique in both research and industrial settings for ensuring the quality and performance of micro-optical elements. The proposed system may be utilized to inspect the quality of micro-optical components like micro-lenslet arrays. In this section, the quantitative phase imaging capability of the proposed system is experimentally demonstrated on the phase objects, including the refractive micro-lenslet array. The phase samples are prepared on the reflecting surface. First, an experiment is performed on the refractive micro-lenslet array (MLA300-14AR-M fabricated from Thorlabs, USA) placed on a mirror. The lens size and the focal length of the micro-lenslet array are 295 μm and 14.6 mm, respectively. A digital hologram is recorded in the presence of the micro-lenslet array with the use of a microscope objective lens of 20× magnification. Figure 4a shows the retrieved wrapped phase obtained from the recorded digital hologram. This phase distribution is wrapped (−π, π), and to obtain a continuous phase distribution, an unwrapped phase algorithm is used. The obtained 2D and pseudo-3D unwrapped phase maps are depicted in Figure 4b and Figure 4c, respectively. The thickness of the micro-lenslet array is measured using the following relationship:

(1) $$h=\frac{∆\emptyset \times \lambda }{2\pi \times ∆n}$$

where ∆∅ is the unwrapped phase, λ is the wavelength used in the experiment (632.8 nm), and $∆n=n_{s}-n_{m}$, with *n_s_* and *n_m_* being the refractive indices of the sample and medium, respectively.

Figure 4d depicts the pseudo-3D thickness profile of the micro-lenslet array obtained using Equation (1), from the measured unwrapped phase and using other known parameters. Moreover, the obtained phase information may further be used for the evaluation of other parameters, including the sag height, diameter, radius of curvature, and focal length of the lens [51]. The measured sag height is 1.74 μm. The radius of curvature (*ROC*) of the lens is calculated by the following relationship:

(2) $$h=ROC-\sqrt{{ROC}^{2}-r^{2}}$$

where *r* is the radius of the lens, which is estimated by counting the number of pixels and pixel sizes occupied by the lens in the obtained phase image. Furthermore, the focal length of the lens is calculated by the following relationship:

(3) $$f=\frac{ROC}{n-1}$$

The experimentally measured values of *ROC* and *f* are 6.466 mm and 14.15 mm, respectively. Based on the findings of the experiments, it is possible to deduce that the system may be used to precisely measure different micro-optical components. Furthermore, the lens height, *ROC*, focal length, aberration coefficients, topographic maps, surface roughness, and refractive index of these micro-optical components may be quantitatively assessed. So, the proposed DHM plays a significant role in the testing and characterization of micro-optics, offering several advantages over traditional microscopy and measurement techniques.

### 3.4. Quantitative Phase Imaging of Biological Cells

**Figure 5:**
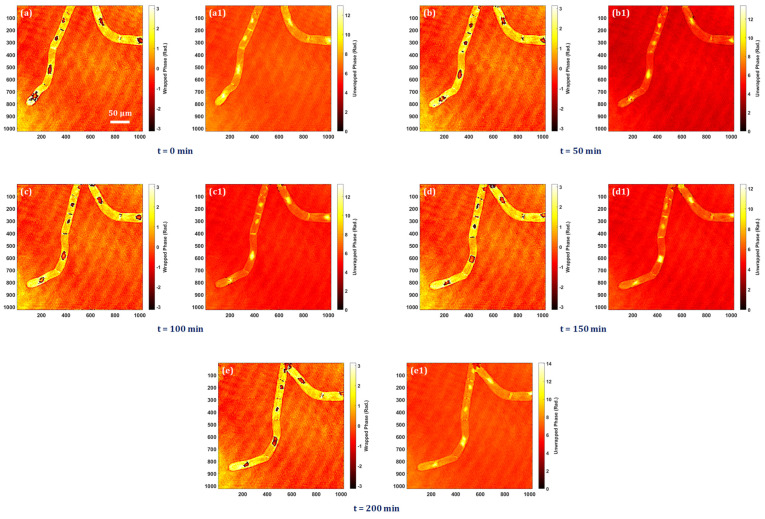
Experimental results of tobacco plant cells at different instants of time: (**a**,**a1**) at t = 0 min, (**b**,**b1**) at t = 50 min, (**c**,**c1**) at t = 100 min, (**d**,**d1**) at t = 150 min, and (**e**,**e1**) at t = 200 min.

The bio-imaging application capability of the system is presented by performing the experiment on tobacco plant cells with a microscope objective lens of 20× magnification. The sample is prepared on a silver mirror as stated above, and the digital holograms are recorded every five minutes over four hours in order to observe the time-lapse quantitative phase information of the cells and nuclei throughout this time period. Time-lapse quantitative phase information is crucial for unraveling the complexities of cellular behaviors, providing a dynamic and quantitative perspective that is essential for advancing our understanding of cell biology, disease processes, and therapeutic interventions. The experimentally obtained quantitative phase images of tobacco plant cells at different instants of time are shown in Figure 5a–e. Figure 5a–e shows the wrapped phase maps, and their corresponding unwrapped phase maps are shown in Figure 5(a1–e1). Visualization (see supplementary material) shows the time-lapse retrieved wrapped phase imaging video of tobacco plant cells captured at five-minute intervals. In this visualization, it is possible to see the nuclei dynamics as well as the quantitative analysis of dynamic morphological changes in cells and nuclei.

## 4. Discussion

DHM serves as a powerful tool with broad applications in biological research and optical metrology of micro-optics. Its ability to provide quantitative, high-resolution phase information makes it valuable for advancing our understanding of biological cells and ensuring the quality and performance of optical components in various industries. Therefore, the field of DHM and related techniques has seen continuous advancements. Recent trends indicate a continued evolution of DHM technologies toward higher performance, broader applicability, and increased ease of use. As research in this field progresses, we can expect further innovations and applications that leverage the unique capabilities of DHM for advanced imaging and analysis. In this line of research, we have made a significant advancement in this field by proposing a single-shot, common-path, and wide FOV DHM that can be used for both types of objects, namely reflective and (semi-) transparent, without any modifications. Therefore, the system can be utilized for the investigations of different types of objects. The optical design is inspired by our previous works [39,40,41]. However, it has some differences compared to these systems. In Ref. [39], we employed dual-illumination beams to the object and recorded a multiplexed digital hologram in order to measure out-of-plane and in-plane displacements simultaneously. In Ref. [40], a wedge plate is used to generate the clean reference beam, and the system is used for displacement/vibration measurements. In Ref. [41], an additional beam splitter is used for recording double FOV in a multiplexed digital hologram.

The system overcomes the limitations of existing common-path DHMs where the reference beam is generated from the object beam by employing a specialized optical component, e.g., in Ref. [21], and then spatially filtering it by a pinhole. This process is a little difficult as it requires a precise alignment of the pinhole and sufficiently reduces the laser intensity. Furthermore, the second category of common-path DHMs [29,30,48,49], where a clean portion of the object serves as a reference beam, has the limitations of reduced FOV and is appropriate for sparse samples only. These limitations of both types of common-path DHMs are overcome in the proposed system.

Owing to the common-path geometry, the system shows high temporal phase stability, as experimentally measured in Section 3.1. Such high stability of an optical system is desired in the measurement of small cell thickness fluctuation. The system shows great potential for the quantitative phase imaging of microstructures and biological cells, as experimentally demonstrated in the previous section (Section 3.2, Section 3.3 and Section 3.4). The imaging and optical inspection and investigation capabilities of the proposed system are verified by conducting several experiments on different kinds (reflective surfaces, micro-optics, and biological cells) of objects. The system shows great imaging capability on a reflective surface, a USAF resolution chart, by extracting the amplitude and phase information from a single recorded digital hologram, as presented in Section 3.2. We adopted a computational method, the PCA-based phase compensation method proposed by Zuo et al. [52], for extracting the amplitude and phase information from a single digital hologram. Then, the quantitative inspection of a micro-lenslet array is carried out in Section 3.3. Several physical parameters of the micro-lenslet array are measured from the obtained phase information of the array. These parameters are sag height, diameter, radius of curvature, focal length, etc. The experimentally measured values of these parameters are in good agreement with their true values. Furthermore, the time-lapse quantitative phase imaging of tobacco plant cells is experimentally investigated by the proposed DHM system, and the experimental results are presented in Section 3.4. The obtained quantitative phase imaging of the tobacco plant cells demonstrated the use of the proposed DHM system in the field of biomedical imaging. The obtained phase information of the plant cells can further be used for the investigations of other biophysical parameter evaluations.

This facilitates the possibilities of computation and evaluation of several biophysical parameters of biological cells as well as the measurement of several valuable physical parameters of micro-optics and microstructures. We can say that the system has the potential to contribute to various research areas, including cell biology, biophysics, and medical research, where understanding cellular dynamics and morphology is crucial for advancing our knowledge of health and disease. Also, its applications range from inspecting microscopic structures to evaluating the overall quality and performance of manufactured components. The precision and efficiency of the proposed system contribute to improving manufacturing processes, reducing defects, and ensuring the reliability of industrial products. We anticipate that this proposed system will be useful in life science and industrial inspection.

However, the proposed system has some limitations despite its many advantages. One limitation is the reduction of light throughput due to the use of several beam splitters. However, this issue is exacerbated in the case of incoherent holography, particularly when the fluorescent light from the object passes through several optical components (e.g., beam splitters). In our experiments, we used a 20 mW laser and did not observe any low-light issues. Compared to the current optical framework, an extra beam splitter is utilized in our other DHM system with double FOV imaging capability [41], and we did not detect any low-light issues. Thus, we can conclude that this optical design does not have a low-light issue for a laser source with a power of 20 mW or more. Another issue may be multiple reflections. This issue of multiple reflections can be overcome by employing a wedge plate instead of the beam splitter. The wedge angle should be chosen so that the reflection from one surface of the wedge plate (either front or rear) cannot incident on the image sensor’s faceplate. Conversely, if the alignment is not perfect, there may be several reflections off the sample surface, or the optical components, particularly beam splitters, will produce undesired interference patterns.

## 5. Conclusions

We introduced a new configuration of a single-shot wide field-of-view common-path off-axis reflective DHM for quantitative phase imaging and inspection of engineered surfaces. The optical design of the proposed system consists of a plate beam splitter to generate a reference beam. The system provides a very high temporal phase stability of ~0.00046, which is >43 times higher than a two-channel DHM system. The system neither requires special optical components nor complicated optical designs to achieve the common-path geometry; therefore, it is much easier to couple it with other imaging modalities. The proposed system with a compact design, single-shot acquisition, common-path configuration, and utility for both reflective as well as (semi) transparent objects will be a crucial imaging tool in a wide range of applications. The topography of the opaque object and quantitative phase imaging capability of the system are experimentally demonstrated by retrieving the complex-field imaging of the USAF resolution target and living tobacco plant cells, respectively. The system is switchable from reflection mode to transmission mode and vice versa, enabling its use for investigating different types of samples, and therefore, it has the potential to be utilized in many kinds of research fields, including life science and industrial inspection.

## Data Availability

The data that support the findings of this study are available from the corresponding author upon reasonable request.

## Supplementary Materials

The following supporting information can be downloaded at: https://www.mdpi.com/article/10.3390/s24030720/s1, See the supplementary material for visualization of the time-lapse retrieved wrapped phase imaging video of tobacco plant cells captured five-minute intervals.
